# A multicenter randomized clinical trial investigating the cost-effectiveness of treatment strategies with or without antibiotics for uncomplicated acute diverticulitis (DIABOLO trial)

**DOI:** 10.1186/1471-2482-10-23

**Published:** 2010-07-20

**Authors:** Çağdaş Ünlü, Niels de Korte, Lidewine Daniels, Esther CJ Consten, Miguel A Cuesta, Michael F Gerhards, Anna AW van Geloven, Edwin S van der Zaag, Joost AB van der Hoeven, Rutger Klicks, Huib A Cense, Rudi MH Roumen, Quirijn AJ Eijsbouts, Johan F Lange, Paul Fockens, Corianne AJM de Borgie, Wilem A Bemelman, Johannes B Reitsma, Hein BAC Stockmann, Bart C Vrouenraets, Marja A Boermeester

**Affiliations:** 1 Department of Surgery, Sint Lucas Andreas Hospital Amsterdam, the Netherlands; 2 Department of Surgery, Academic Medical Center Amsterdam, the Netherlands; 3 Department of Surgery, Kennemer Gasthuis, Haarlem, the Netherlands; 4 Department of Surgery, Meander Medisch Centrum Amersfoort, the Netherlands; 5 Department of Surgery, VU Medical Center Amsterdam, the Netherlands; 6 Department of Surgery, Onze Lieve Vrouwe Gasthuis Amsterdam, the Netherlands; 7 Department of Surgery, Tergooi Hospital Hilversum, the Netherlands; 8 Department of Surgery, Gelre Hospital Apeldoorn, the Netherlands; 9 Department of Surgery, Albert Schweitzer Hospital Dordrecht, the Netherlands; 10 Department of Surgery, Boven IJ Hospital Amsterdam, the Netherlands; 11 Department of Surgery, Rode Kruis Hospital Beverwijk, the Netherlands; 12 Department of Surgery, Maxima Medisch Centrum Veldhoven, the Netherlands; 13 Department of Surgery, Spaarne Hospital Hoofddorp, the Netherlands; 14 Department of Surgery, Erasmus Medical Center Rotterdam, the Netherlands; 15 Department of Gastroenterology, Academic Medical Center Amsterdam, the Netherlands; 16 Department of Clinical Epidemiology and Biostatistics, Academic Medical Center Amsterdam, the Netherlands

## Abstract

**Background:**

Conservative treatment of uncomplicated or mild diverticulitis usually includes antibiotic therapy. It is, however, uncertain whether patients with acute diverticulitis indeed benefit from antibiotics. In most guidelines issued by professional organizations antibiotics are considered mandatory in the treatment of mild diverticulitis. This advice lacks evidence and is merely based on experts' opinion. Adverse effects of the use of antibiotics are well known, including allergic reactions, development of bacterial resistance to antibiotics and other side-effects.

**Methods:**

A randomized multicenter pragmatic clinical trial comparing two treatment strategies for uncomplicated acute diverticulitis. I) A conservative strategy with antibiotics: hospital admission, supportive measures and at least 48 hours of intravenous antibiotics which subsequently are switched to oral, if tolerated (for a total duration of antibiotic treatment of 10 days). II) A liberal strategy without antibiotics: admission only if needed on clinical grounds, supportive measures only. Patients are eligible for inclusion if they have a diagnosis of acute uncomplicated diverticulitis as demonstrated by radiological imaging. Only patients with stages 1a and 1b according to Hinchey's classification or "mild" diverticulitis according to the Ambrosetti criteria are included. The primary endpoint is time-to-full recovery within a 6-month follow-up period. Full recovery is defined as being discharged from the hospital, with a return to pre-illness activities, and VAS score below 4 without the use of daily pain medication. Secondary endpoints are proportion of patients who develop complicated diverticulitis requiring surgery or non-surgical intervention, morbidity, costs, health-related quality of life, readmission rate and acute diverticulitis recurrence rate. In a non-inferiority design 264 patients are needed in each study arm to detect a difference in time-to-full recovery of 5 days or more with a power of 85% and a confidence level of 95%. With an estimated one percent of patients lost to follow up, a total of 533 patients will be included.

**Conclusion:**

A clinically relevant difference of more than 5 days in time-to-full recovery between the two treatment strategies is not expected. The liberal strategy without antibiotics and without the strict requirement for hospital admission is anticipated to be more a more cost-effective approach.

**Trial registration:**

Trial registration number: NCT01111253

## Background

Prevalence of diverticular disease increases with age, from less than 10% in people younger than age 40 to 50-66% in octogenarians, with similar frequency in men and women. Approximately three quarters of patients with diverticulosis remain asymptomatic throughout their lifetime. Asymptomatic disease is often an incidental finding during imaging or endoscopy for suspicion of colonic disorders. Of the 25% of patients who develop symptomatic diverticular disease, approximately three quarters develop diverticulitis [1,2]. Of all patients with diverticulitis, 75% have mild acute disease only and 25% develop complicated disease [3]. All and all about 5% of patients with diverticulosis will undergo an episode of complicated diverticulitis.

The cause of colonic diverticular disease has not yet been conclusively established. Epidemiologic studies have demonstrated associations between diverticulosis and diets that are low in dietary fiber and high in refined carbohydrates. Low intake of dietary fiber results in less bulky stools retaining less water and altering gastrointestinal transit time. These factors could increase intracolonic pressure (development of pressure zones that create diverticula alongside the vasa recta), and make evacuation of colonic contents more difficult [4]. Other factors that have been associated with an increased risk of diverticular disease include physical inactivity, constipation, obesity, smoking, and treatment with non-steroidal anti-inflammatory drugs [5,6].

Although much has been learned about the development of diverticula, less is known about the pathogenesis of diverticular inflammation. As discussed earlier, a minority of patients with diverticulosis will develop symptomatic disease. Initial theories of diverticulitis focused on ideas about the pathogenesis of appendicitis; a diverticulum lumen becomes obstructed by a faecolith leading to increased intradiverticular pressure and eventually causing inflammation. Interest has been generated in the role of altered peridiverticular colonic flora and low-grade chronic inflammation leading to periods of symptomatic disease, similar to periods of exacerbation and remission in inflammatory bowel disease [7].

The classical clinical presentation of diverticulitis in the western world includes left lower quadrant abdominal pain, tenderness, low-grade fever and leucocytosis. However, clinical features can be quite variable. Leucocytosis may only be present in 45-65% of the patients, and low-grade fever may be present in only 21% [8].

For a reliable diagnosis additional imaging is usually necessary. Computed tomography (CT) is recommended as initial radiological examination. Positive findings in ultrasound (US) are equally accurate in the diagnosis of diverticulitis. However CT has an advantage in excluding alternative diagnoses and visualising complications of acute diverticulitis needing intervention. For both US and CT, sensitivity is as high as 90%, with a specificity of up to 99% for CT [9].

The severity of diverticulitis is often graded with the use of modified Hinchey's criteria, based on CT imaging and on preoperative findings [10,11]. The Ambrosetti's criteria is based only on CT imaging, classifying in "mild" and "severe" diverticulitis. This classification system does not take into account the effects of coexisting conditions on disease severity or outcome [12]. (Table 1) Stage II disease is related to a large (> 5 cm) collection of pus, which is at distance (in the pelvis or the abdomen) of the sigmoid colon [10]. Stage II usually requires percutaneous drainage, while stages III and IV diverticulitis usually request surgery.

**Table 1:** Hinchey classification and modified Hinchey classification of acute diverticulitis [10,12]

Hinchey	Modified Hinchey
	0 Mild clinical diverticulitis

I Pericolic abscess or phlegmon	1a Colonic wall thickening/Confined pericoloc inflammation
	
	Ib Confined small (< 5 cm) pericolic abscess

II Pelvic, intraabdominal, or retroperitoneal abscess	II Pelvic, distant intraabdominal, or retroperitoneal abscess

III Generalized purulent peritonitis	III Generalized purulent peritonitis

IV Generalized fecal peritonitis	IV Fecal peritonitis

Conservative treatment of mild diverticulitis usually includes careful observation, restriction of oral intake, administration of intravenous fluids, and most patients receive antibiotic therapy. The majority of patients with mild diverticulitis improve with these conservative measures. Less than 10% need percutaneous or operative treatment for disease progression and/or complications [13,14].

It is, however, uncertain whether patients with acute diverticulitis benefit from antibiotics, since evidence from prospective studies or randomized trials is lacking. In a recent review antibiotics are considered mandatory in the treatment of mild diverticulitis [15]. This advice lacks evidence and is based on experts' opinion only. Anaerobes are commonly isolated organisms in acute diverticulitis. Gram-negative aerobes, especially Escherichia coli, and facultative gram-positive bacteria, such as streptococci, are often cultured as well [16]. Therefore, broad-spectrum antibiotics are advised. Which antibiotic regimen should be used in diverticulitis is unclear [17,18]. There is scarse evidence that oral antibiotics are as effective as intravenous antibiotics [19].

Only one study has investigated the use of antibiotics in the treatment of acute uncomplicated diverticulitis. In a retrospective study by Hjern et al [20], there was no significant benefit from antibiotics in the treatment of mild diverticulitis. However, this study was hampered by selection bias due to its retrospective design and small patient groups.

Moreover, there is major discrepancy in the use of antibiotics between countries in Northwest Europe and other countries, including the United States and United Kingdom. In the Netherlands and Scandinavian countries antibiotic use for this disease is less common compared to these other countries, where antibiotics are considered mandatory. A Dutch survey showed that many gastro-enterologists prescribed antibiotics in the treatment of acute diverticulitis, but only a minority of Dutch surgeons did so [21]. In contrast, all UK surgeons responding to a survey prescribed antibiotics in the initial treatment of diverticulitis and 43% of them even for 7 days after hospital discharge [22].

Six professional organisations have issued formal guidelines concerning the use of antibiotics in uncomplicated diverticulitis. Five of these guidelines advice the use of antibiotics. (Table 2) [23-28]. Patients should start with intravenous antibiotics and after improvement within 2-4 days, oral antibiotics are continued to complete a 7-10 days treatment regimen. In the Netherlands, the Dutch Antibiotic Policy Committee considers antibiotics not primarily indicated in the treatment of uncomplicated diverticulitis [28].

**Table 2:** Published guidelines and practise parameters

Organization	Year	Antibiotics Recommended	Original research cited	Which antibiotics	Original research cited	Route of administering	Original research cited
**American College of Gastroenterology**[23]	1999	Yes	None	Covering both Gram negative and anaerobes	Kellum [15]	Oral or intravenous, depending on clinical status	None

**European Association for Endoscopic Surgery**[25]	1999	Yes	None	Ciprofloxacin And Metronidazol	None	Oral or intravenous, depending on clinical status	None

**American Society of Colon and Rectal Surgeons**[24]	2006	Yes	None	Covering both Gram negative and anaerobes	Kellum [15]	Oral or intravenous, depending on clinical status	None

**Society of Surgery of the Alimentary Tract**[26]	2007	Yes	None	Broad spectrum antibiotics	None	Oral or intravenous, depending on clinical status	None

**World Gastroenterology Organization**[27]	2007	Yes	None	Covering both Gram negative and anaerobes	None	Oral or intravenous, depending on clinical status	None

**SWAB**[28]	2009	No, not primarily	None	Broad spectrum antibiotics	None	Oral or intravenous, depending on clinical status	None

Adverse effects of antibiotics are well known, such as allergic reactions and development of antibiotic resistance of bacterial species. The frequency of toxicodermia is 7-8% with the use amoxicillin, allergy reactions are accounted for in 1% of the patients, and the incidence of anaphylactic shock is 0,01-0,04% with the use of penicillin. Therefore, efforts are made to minimize the use of antibiotics in various fields in clinical medicine [29].

The lack of evidence for its use necessitates a scientific judgement of the role of antibiotics in the treatment of uncomplicated diverticulitis. Therefore, we initiated a randomized multicenter trial to investigate the effect of antibiotics on disease course in patients with mild acute diverticulitis.

## Methods/Design

### Objective

The main goal of the present study is to establish whether antibiotics are necessary in the primary treatment of acute mild diverticulitis, and whether a more liberal strategy without initial antibiotics is more cost-effective with respect to time-to-full recovery.

In daily practice there is an ongoing discussion about the relative benefits and disadvantages of a more conservative treatment strategy embracing the use of intravenous antibiotics. This strategy needs hospital admission and is, at least at the start, an in-hospital treatment regimen. A more liberal strategy, without antibiotics and without the strict requirement of hospital admission, may lead to a shorter hospital stay and reduced costs without compromising outcome.

Our hypothesis is that in uncomplicated (mild) acute diverticulitis, a liberal strategy treatment without antibiotics is a more cost-effective approach than conservative treatment strategy with hospital admission and antibiotics, outcome is measured by time-to-full recovery as primary outcome and diverticulitis-associated complication rates and patient well-being as secondary outcome.

### Study population

**Figure 1:**
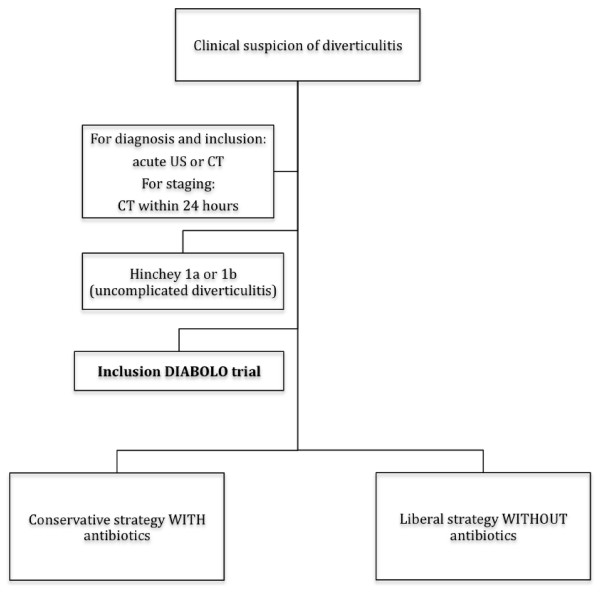
**Study flow chart**.

Inclusion criteria:

1. Only left-sided and primary (first attack) mild acute diverticulitis.

2. Diagnosis of diverticulitis by US and conditional CT. Diverticulitis-positive US findings are sufficiently accurate compared to CT findings [9]. In diverticulitis-negative US findings in clinically suspected patients, immediate i.v. contrast-enhanced CT is mandatory for confirmation of diverticulitis and exclusion of other pathology.

3) Staging of diverticulitis by CT. CT is needed for all patients for Hinchey/Ambrosetti classification (which is a CT-based classification system). In diverticulitis-positive US findings CT has to be performed within 24 hours. Staging diverticulitis is defined according the modified Hinchey/Ambrosetti staging. Only modified Hinchey stages 1a and 1b (1a Colonic wall thickening/Confined pericolic inflammation, 1b Confined small pericolic abscess) and Ambrosetti's "mild" diverticulitis stage are included. Figure 1 depicts a flow chart, showing the inclusion criteria and the steps after inclusion [10-12].

4. Informed consent.

Exclusion criteria are summarized in Table 3.

**Table 3:** Exclusion criteria

1. Previous radiological (US and/or CT) proven episode of diverticulitis;
2. US and/or CT suspicion of colonic cancer

3. Inflammatory bowel disease (ulcerative colitis, Crohn's disease);

4. Hinchey stages 2, 3 and 4 or "severe" diverticulitis according to the Ambrosetti criteria, which require surgical or percutaneous treatment;

5. Other disease with expected survival of less than 6 months;

6. Contraindication for the use of the study medication (e.g. patients with advanced renal failure or allergy to antibiotics used in this study);

7. Pregnancy;

8. ASA (American Society of Anesthesiologists) classification > III;

9. Immunocompromised patient; (i.e., haematological malignancies, AIDS patients with low CD4+ counts, transplantation, chemotherapy, splenectomy, long-term corticosteroid use and genetic disorders such as severe combined immunodeficiency.

10. Clinical suspicion of bacteraemia (i.e. sepsis);

11. The ability of reading/understanding and filling in the questionnaires.

12. Antibiotic use in the 4 weeks prior to inclusion

### Study outline

Patients will be randomly allocated to one of the following two treatment strategies: Conservative strategy including immediate antibiotic treatment or liberal strategy without antibiotics (supportive measures only). (Table 4).

**Table 4:** Treatment strategies

Conservative strategy with antibiotics	Liberal strategy without antibiotics
- Hospital admission;	- Admission only if discharge criteria are not met;

- Intravenous fluids and at least 48 hours of intravenous antibiotics and subsequently switch to oral antibiotics if tolerated (otherwise continuation i.v.) to complete a full 10-day treatment duration;	- No initial antibiotics;

- Adequate pain relief (VAS < 4);	- Adequate pain relief (VAS < 4);

- Oral intake as tolerated;	- Oral intake as tolerated;

- Daily monitoring.	- Daily monitoring when admitted to the hospital;

-Self monitoring at home after discharge -Out patient follow-up at regular intervals	- Self monitoring at home. - Out patient follow-up at regular intervals

In the conservative strategy, the use of antibiotics will be intravenously for at least 48 hours after which route of administration can be switched to orally if tolerated. Hospital admission in the liberal strategy is needed for patients with nausea and vomiting, in need of intravenous fluids or for patients with excessive pain not properly reacting to oral pain medication.

The interval between start of symptoms of the patient and administration of antibiotics will be registered. Also the period after inclusion and the actual first administration of antibiotics will be registered.

In both strategies CT is repeated in case of clinical deterioration. For patients in the liberal strategy treatment arm, clinical deterioration and/or proven subsequent complicated diverticulitis and/or other infectious foci (e.g., pneumonia, infections) may dictate start of antibiotic treatment, instigated by the treating physician. Criteria to start antibiotics in the liberal arm are temperature > 39°C, positive blood cultures and clinical suspicion of bacteraemia (i.e. sepsis). Criteria for sepsis are set by the American College of Chest Physicians and the Society of Critical Care Medicine. Two or more symptoms are required: Body temperature < 36°C or > 38°C, heart rate higher than 100 beats a minute, respiratory rate higher than 20 breaths a minute and white blood cell count < 4 × 10^9 ^or > 12 × 10^9 ^cells/L [31]. Also another infectious focus (e.g., pneumonia, urinary tract infections) may dictate start of antibiotic treatment, instigated by the treating physician.

The following discharge criteria are applied in both strategies: normal diet (defined by tolerating solid food and more than 1L of fluid orally), temperature < 38.0°C, VAS (Visual Analoge Score) pain score < 4 (with paracetamol only), self support as compared to the pre-illness level, and acceptance by the patient.

All outpatients will daily monitor and register their body temperature. Written and oral instructions at discharge are given, and relevant telephone numbers and contact information will be provided. In case of fever above 38˚C, progression of pain above a VAS of 4 or other clinical signs of deterioration, patients can contact the hospital or emergency department immediately.

#### Antibiotics

For the choice and duration of antibiotics the practice guidelines of the Dutch Antibiotic Policy Committee [28] and the American Society of Colon and Rectal Surgeons [30] are followed. In both guidelines, a minimum of 7-14 days of broad-spectrum antibiotics is advised. In the present study amoxicillin-clavulanic acid is chosen as broad-spectrum antibiotic; duration of antibiotic treatment is 10 days. The dosage scheme for the study drug is 1200 mg i.v. 4 times daily with subsequent oral administration of 625 mg 3 times daily. In case of allergy (known or newly diagnosed), a switch will be made to the combination of ciprofloxacine and metronidazole; ciprofloxacine 2 times a day 400 mg i.v. and metronidazol 3 times daily 500 mg, with oral doses of ciprofloxacine being 500 mg 2 times a day and of metronidazol 3 times a day 500 mg.

### Endpoints

The primary endpoint is time-to-full recovery within a follow-up period of 6 months. Full recovery is defined by the following criteria: discharged from the hospital (out-patient), normal diet (defined by tolerating solid food and more than 1L of fluid orally), temperature < 38.0°C, and VAS pain score < 4, no use of daily pain medication or back to pre-illness pain medication use, and resuming to pre-illness working activities; as assessed by questionnaires and out-patient clinic visits.

The secondary endpoints are: proportion of patients who develop complicated diverticulitis require surgery or non-surgical intervention; number of days outside the hospital in a 6 months period; direct and indirect medical costs at 6 months follow-up; occurrence of complicated diverticulitis defined as abscess, perforation, stricture and/or fistula; predefined side-effects of initial antibiotic treatment (e.g. antibiotic resistance/sensitivity pattern, allergy); morbidity (e.g. pneumonia, myocardial infarction, urinary tract infection); mortality; readmission rate within 6 months and acute diverticulitis recurrence rates at 12 and 24 months follow-up. Changes in health status and valuation over time will be measured using generic and disease specific quality of life questionnaires (Euro-Qol 5D, Short Form 36 (SF-36) and the Gastro-Intestinal Quality of Life Index (Giqli)) on admission and after 3, 6, 12 and 24 months.

A recurrence is defined as ultrasound- or CT-proven acute diverticulitis after complete resolution of symptoms more than 1 month after initial discharge from hospital. If a patient dies during follow-up, the reason for death will be recorded as related or unrelated to diverticular disease.

### Randomization

Computerized block randomization for allocation of treatment group, stratified for center and for Hinchey 1a and 1b, will take place after all inclusion and exclusion criteria have been verified and informed consent has been obtained. A standardized case record from (CRF) will be used. This CRF is partially web-based via a secured internet module. A minimum of 10% of the CRF data will be verified with source data by an independent audit.

### Sample size calculation and date analysis

A non-inferiority design was chosen. Time-to-full recovery in the liberal strategy arm must not exceed a clinically relevant difference of more than 5 days compared with the conservative strategy. When this condition is fulfilled, the potential advantages of the liberal (non-antibiotic) strategy become dominant: patient well being when the need of hospital admission can be avoided, less costs, less antibiotic resistance and less other side effects. The study must have the power (superiority) to detect a difference in time-to-full recovery of 5 days.

The median time-to-full recovery is 21 days based on the National Dutch Hospital Registry data with an average of 7 days admission and an assumed additional median 14-day out-patient period to full recovery. To reject the null-hypothesis of a difference in time-to-full recovery of 5 days or less, using a time-to-event analysis with a power of 85% at a confidence level of 95%, an accrual period of 730 days and a follow-up period of 180 days, at least 264 patients need to be included in each treatment arm. With an estimated one percent of the trial patients lost to follow-up, a total 533 patients is needed.

The primary endpoint is time-to-full recovery. Kaplan-Meier curves depicting the proportion of patients with full recovery since randomisation will be constructed for both strategies. The log rank test will be used to test for superiority of one strategy compared with the other. Testing for non-inferiority will be done by calculating the hazard ratio for the liberal strategy compared with the conservative strategy using Cox regression. We will calculate a one-sided 95% confidence interval for this ratio to determine whether it reaches outside the hazard ratio belonging to an equivalence limit of a difference of 5 days in median survival time.

For other endpoints data will be compared by the Student's t test, Wilcoxon rank sum test, Chi square test or Fischer exact test as appropriate. In superiority tests a two-tailed P value ≤ 0.05 will be considered statistically significant, whereas one-sided tests will be performed in non-inferiority testing. The main analyses will be based on the intention to treat principle. Predefined subgroup analyses to investigate whether treatment effects are different in subgroups will be performed for Hinchey classification 1a versus 1b and for participating center.

### Cost analysis

All related costs will be estimated based on the actual input terms of resource use and personnel in the 6-month follow-up period after randomization. For all cost-items such as hospital admission, medication used, diagnostic tests, unit costs will be derived from the Dutch costing manual or determined in cooperation with the hospital administration. Direct medical costs will be recorded in the case record forms. Indirect costs arising from losses in productivity will be assessed by means of the Health and Labor questionnaire and will be calculated by means of the friction cost method.

### Economic evaluation

The economic evaluation will be performed from a societal perspective as a cost-effectiveness and cost-utility analysis. The main analyses include costs per day reduction to achieve full recovery and costs per QALY gained. Additional sensitivity analyses, regarding differences in possible subgroups, will be performed.

### Safety monitoring

Adverse events are defined as any undesirable experience occurring to a subject during a clinical trial, whether or not considered related to the investigational drug. All adverse events reported spontaneously by the subject or observed by the investigator or his staff will be recorded. A serious adverse event (SAE) is any untoward medical occurrence or effect that at any dose results in death; is life threatening (at the time of the event); requires hospitalization or prolongation of existing inpatients' hospitalization; results in persistent or significant disability or incapacity; is a congenital anomaly or birth defect; is a new event of the trial likely to affect the safety of the subjects, such as an unexpected outcome of an adverse reaction, major safety finding from a newly completed animal study, etc. All SAEs will be reported to the accredited Medical Ethical Committee (MEC) that approved the protocol, according to the requirements of that MEC.

Suspected unexpected serious adverse reactions (SUSAR) are all untoward and unintended responses to an investigational product related to any dose administered.

Unexpected adverse reactions are adverse reactions, of which the nature, or severity, is not consistent with the applicable product information.

The sponsor will report expedited the following SUSARs to the MEC; SUSARs that have arisen in the clinical trial that was assessed by the MEC; SUSARs that have arisen in other clinical trial of the same sponsor and with the same medicinal product, and that could have consequences for the safety of the subjects involved in the clinical trial that was assessed by the MEC. The remaining SUSARs are recorded in an overview list (line-listing) that will be submitted once every half year to the MEC. This line listing provides an overview of all SUSARs from the study medicine, accompanied by a brief report highlighting the main points of concern.

The sponsor will report expedited all SUSARs to the competent authority, the Medicine Evaluation Board and the competent authorities in other Member States. The expedited reporting will occur not later than 15 days after the sponsor has first knowledge of the adverse reactions. For fatal or life threatening cases the term will be maximal 7 days for a preliminary report with another 8 days for completion of the report. There is no need to break any code in case of a SUSAR because due to the nature of the study in which neither participant nor treating physician are blinded.

In addition to the expedited reporting of SUSARs, the sponsor will submit, once a year throughout the clinical trial, a safety report to the accredited MEC, competent authority, Medicine Evaluation Board and competent authorities of the concerned Member States. This safety report consists of: a list of all suspected (unexpected or expected) serious adverse reactions, along with an aggregated summary table of all reported serious adverse reactions, ordered by organ system, per study; a report concerning the safety of the subjects, consisting of a complete safety analysis and an evaluation of the balance between the efficacy and the harmfulness of the medicine under investigation.

An independent data and safety monitoring committee will evaluate the progress of the trial and will examine safety parameters at regular intervals (every 25 patients). The committee can unblind the data whenever deemed necessary based on reported adverse events. All involved physicians will repetitively be asked to report any potential adverse events caused by the study protocol. These adverse events will be listed and discussed with the monitoring committee. The monitoring committee can ask for a full report in order to discuss a specific adverse event. A copy of this report will be send to the central ethics board and to the involved physicians. All deceased patients will be evaluated by the safety committee for cause of death and possible trial related serious adverse effects. Every death will be reported to the central ethics board and the local ethics board. The Data Safety Monitoring Board will consist of an epidemiologist/statistician who is the chairman, an independent surgeon and an independent radiologist.

### Ethics

This study is conducted in accordance with the principles of the Declaration of Helsinki and 'good clinical practice' guidelines. The Medical Ethical Committee of the Academic Medical Center in Amsterdam has approved the protocol. The Ethical Committees of the participating centers is applied for local feasibility. Prior to randomization, written informed consent will be obtained from all patients.

## Discussion

Diverticular disease is the most common disease of the colon being found in every 1 of 3 people over the age of 60 years. The overall prevalence of diverticular disease during endoscopy is 27% [32]. A recent task force convened by the American Gastroenterological Association confirmed that diverticular disease is a major clinical problem. Diverticular disease is fifth in the list of digestive diseases in terms of total costs [33]. Hospital admission rates for colonic diverticulitis have increased in the last decades. In the United States the population-adjusted numbers of domestic admissions for acute diverticulitis increased by 26% [34].

Over the last decade there have been efforts made to minimize the prescription of antibiotics in various fields in clinical medicine. Patients with appendiceal inflammatory masses or acute cholecystitis are not treated primarily by antibiotics. This is also true for community-acquired infections, such as acute otitis media, upper respiratory tract infections and in paediatric medicine [35]. Bacterial resistance to antibiotics is a major public-health problem and antibiotic use is being increasingly recognized as the main selective pressure driving this resistance [36,37]. Development of Clostridium-associated diarrhea is however one of the downsides of antibiotic use, and subject of this study. With the use of beta-lactam antibiotics, infection with Clostridium difficile is a potential problem for all hospitalized patients. Clostridium difficile is implicated in 20-30% of patients with antibiotic-associated diarrhea, in 50-70% of those with antibiotic-associated colitis and in more than 90% of those with antibiotic-associated pseudomembranous colitis [38]. Alternatively, there is no evidence or guideline dictating that support anti-anaerobic prophylaxis for hospitalized patients in general. Prophylactic metronidazol to prevent Clostridium-associated diarrhea is not standard practice and is therefore not considered for this trial.

There are some new treatment options for symptomatic diverticular disease under investigation, such as mesalazin and probiotics. For the present randomized trial these treatments were not considered a reasonable alternative. First, these treatment options are not yet widely used and are only applied in the context of clinical trials. These studies have dealt with the treatment of uncomplicated symptomatic diverticular disease, and not with acute diverticulitis. Patients with proven diverticulosis and at least one months of symptoms had been included. These trials have excluded diverticulitis patients [39,40]. Some studies have assessed meselazin in the prevention of recurrent diverticulitis but never as the actual treatment of acute diverticulitis itself [41,42]. Third and foremost, the main topic in daily practice is whether antibiotics are mandatory in the treatment of acute diverticulitis. Until now, no randomized controlled trial has investigated this matter. Before other treatment options become an issue, first the efficacy of antibiotics in diverticulitis needs to be investigated, as this is currently standard practice in many countries.

In the present study we chose for a more pragmatic approach to investigate the effect of antibiotics in the treatment of acute uncomplicated diverticulitis. A clinical randomized trial setting was chosen over a double-blind placebo controlled randomized trial. Our intention is to compare the contemporary treatment strategies in uncomplicated acute diverticulitis. In a pragmatic trial set-up the two possible treatment strategies can be investigated and the outcome will be more applicable in daily practice. In a double-blind placebo controlled trial the effect of antibiotics will be investigated in a more experimental setting were all patients will be admitted and the result will not be applicable to daily practice.

Not all patients with acute diverticulitis have to be admitted to the hospital. In 2005, Mizuki et al showed that outpatient treatment of patients with mild or uncomplicated diverticulitis is safe [43]. For this reason, in the present trial hospital admission is not mandatory in the liberal strategy arm when patients fulfill the 'discharge' criteria at time of study entry. Part of the conservative treatment is hospital admission and intravenous antibiotics as this is common practice. In both arms the same strict criteria for discharge apply.

We decided not to stratify for age, based on the prevalence of diverticulitis in the different age groups and on the latest literature on the outcome of diverticulitis. Diverticulitis occurs in 5-10% by the age of 40 years, in 10-30% by 50 years and in more than 60% by age 80. Recently, Hjern et al reviewed 234 patients with CT-confirmed diverticulitis. The rate of severe diverticulitis observed with CT was lower in the younger patients (2% versus 11.9%; P = 0.025). Surgical management during the first admission was similar in younger patients (2% versus 6.8%; P = 0.271); first episodes of acute diverticulitis being not more aggressive in younger patients [13]. Variables 'severity of disease' (Hinchey 1a (inflammation) versus 1b (plus micro abscesses)) and 'participating and including hospital' were deemed most important with respect to outcome and therefore in need of stratification. Stratification for more than two variables is highly uncommon in randomized control trials.

Right-sided diverticulitis is excluded because of uncertainty about the underlying factors that contribute to right-sided diverticulitis. In literature, a clear distinction is made between left and right-sided diverticulitis. In Western countries, diverticulitis mostly affects the left colon and the incidence of right-sided diverticulitis is estimated to be below 4%. However, in Asia and countries with a high Asian population, diverticular disease of the cecum and the ascending colon is a more widespread disease than the left-sided form of this disease. Sugihara et al reported on 615 Japanese patients with diverticular disease of the colon: 69.8% with right-sided and 15.9% with left-sided and 14.3% both-sided diverticular disease [44]. Left-sided diverticular disease is mainly based on pseudodiverticulae. The pathogenesis is based on a higher intraluminal pressure with consecutive hypertrophy of the colonic wall. In contrast, right-sided diverticulosis, typically is associated with normal intraluminal pressures and a tendency for bleeding rather than perforation, presumably owing to underlying connective tissue abnormality [45]. For the reason of uniformity of study population only left-sided diverticulitis will be included.

## Conclusion

The DIABOLO trial is a multicenter randomized pragmatic trial (trialregister: NL29615.018.09, Clinicaltrial.gov: NCT01111253) comparing the cost-effectiveness of a conservative strategy (with admission and antibiotics) with a liberal treatment strategy (without antibiotics and no strict need for hospital admission) with respect to the primary endpoint time-to-full recovery.

## Competing interests

The authors declare that they have no competing interests.

## Authors' contributions

ÇÜ drafted the manuscript, and ÇÜ, NDK, RJB, BCV and MAB designed the study. All 3D diverticulitis collaboration participated in the definite design of the study during several meetings and critically revised the manuscript. All authors read and approved the final manuscript.

## Pre-publication history

The pre-publication history for this paper can be accessed here:

http://www.biomedcentral.com/1471-2482/10/23/prepub
